# Totally laparoscopic gallbladder-preserving surgery: A minimally invasive and favorable approach for cholelithiasis

**DOI:** 10.3892/etm.2014.2107

**Published:** 2014-12-05

**Authors:** DE-KANG GAO, SHAO-HUA WEI, WEI LI, JIE REN, XIAO-MING MA, CHUN-WEI GU, HAO-RONG WU

**Affiliations:** Department of Hepatobiliary Surgery, The Second Affiliated Hospital of Soochow University, Suzhou, Jiangsu 215004, P.R. China

**Keywords:** gallbladder preservation, cholelithiasis, laparoscopic, cholecystoscope, choledochoscope

## Abstract

The aim of the present study was to investigate the effectiveness of laparoscopic gallbladder-preserving surgery (L-GPS) for cholelithiasis and the feasibility and value of totally laparoscopic GPS (TL-GPS). A total of 517 patients underwent L-GPS, including 365 cases of laparoscopy-assisted GPS (LA-GPS), 143 cases of TL-GPS (preservation rate, 98.3%) and nine conversions to laparoscopic cholecystectomy. The surgeries were all performed by one medical team and the mean operating time was 72 min. All macroscopic calculi were removed through endoscopy. The number of calculi observed in the patients was between one and several dozen; diameters ranged between 0.1 and 2.5 cm. Only three cases of incisional infection were noted in the LA-GPS group and long-term follow-up showed a low recurrence rate of 1.2%. L-GPS is, therefore, an excellent approach to cure cholelithiasis and TL-GPS is a feasible and effective option that could avoid incisional complications.

## Introduction

Cholelithiasis is a global disease. According to a previous study (1), China has a large population of patients with cholelithiasis, including those who have been diagnosed and those to whom the diagnosis is unknown. A large proportion of patients with typical symptoms require a laparoscopic cholecystectomy (LC), which has been regarded as the ‘gold standard’ approach for several years; however, the existence of postcholecystectomy syndromes (2,3) such as chronic abdominal discomfort, alkaline reflux gastritis, dyspepsia and steatorrhea, has focused attention on gallbladder preservation.

Conventional open gallbladder-preserving surgery may remove the calculus from the gallbladder but a long incision is necessary and the absence of a clear macroscopic view may lead to a high incidence of residual stone (4). Laparoscopic gallbladder-preserving surgery (L-GPS), however, is less invasive, and endoscopic surgery makes it possible to observe the inner surface of the gallbladder, which leads to a satisfying cosmetic result and may assist with further exploration and the removal of the existing calculus.

## Materials and methods

### Patients

A total of 517 consecutive patients (402 female and 115 male), aged 21–67 years (average, 36 years), who were diagnosed with cholelithiasis or cholelithiasis with polypus, underwent L-GPS in the Second Affiliated Hospital of Soochow University (Suzhou, China) between April 2009 and March 2014. A total of 143 patients underwent totally laparoscopic GPS (TL-GPS) and 365 patients received laparoscopy-assisted GPS (LA-GPS), with nine conversions to LC. One hundred and fifty-six patients had suffered from acute episodes of biliary colic at least once and 361 patients were asymptomatic or had non-specific upper abdominal discomfort. Sixty-eight patients had between one and three concurrent polypi. For all patients, a bland diet was demanded and a series of preoperative preparations, such as any necessary application of antibiotics or cholaneresis treatments, were adopted. Until clinical symptoms were apparently palliated, an ultrasonic examination was required for disease assessment. The present study was approved by the Ethics Committee of The Second Affiliated Hospital of Soochow University and all procedures were carried out in accordance with the Declaration of Helsinki. Informed, written consent was obtained from all patients (or patients’ families) prior to their inclusion in the study.

### Surgical equipment

Major equipment used included the mini-laparoscope-camera system (Karl Storz Endoscopy-America, Inc., El Segundo, CA, USA), an inflexible cholecystoscope (CHiAO, Guangzhou, China) and a soft choledochoscope (Olympus, Tokyo, Japan). The supporting equipment (CHiAO) included a stone basket, biopsy forceps, electrocoagulation bar and a suction trunk.

### Surgical procedure

Patients underwent surgery under general anesthesia in the supine position, hands by the side. A 6-mm sub-umbilical incision was made, through which a pneumoperitoneum was created with simultaneous CO_2_ insufflation (12–14 mmHg). A trocar was then implanted and the mini-laparoscope was inserted to explore the gallbladder and extrahepatic bile duct.

**Figure 1:**
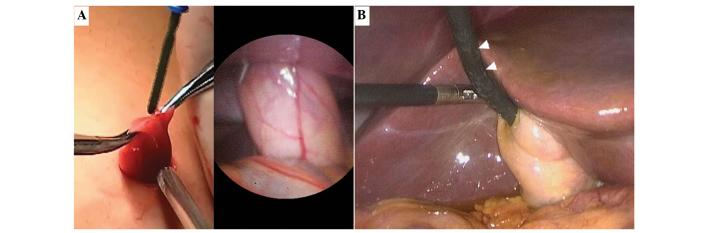
(A) External appearance and laparoscopic view of the gallbladder in LA-GPS. (B) Use of the soft choledochoscope (white arrow) for exploration and calculus removal during TL-GPS. LA-GPS, laparoscopy-assisted gallbladder-preserving surgery; TL-GPS, totally laparoscopic GPS.

**Figure 2:**
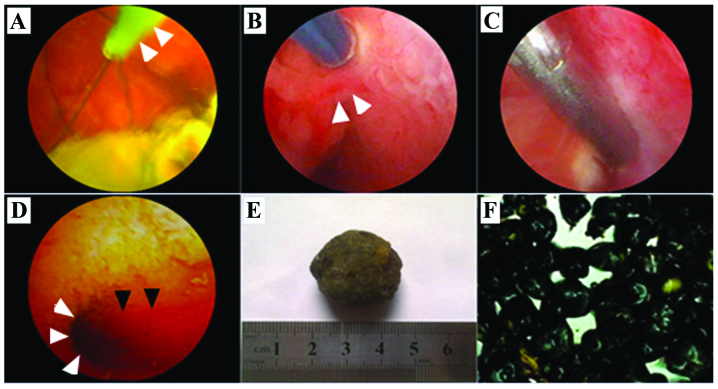
(A) Endoscopic view of calculus removal with a basket (white arrow). (B and C) The appearance of complex or bile pigment gallstones that were removed. (D and E) For polypus removal, the (D) pre-electric coagulation on the polypus pedicle (white arrow) was necessary prior to (E) use of biopsy forceps. (F) Spurting bile (black arrow) from the opening of the cystic duct (white arrow) during extended exploration.

For LA-GPS, a 2.0-cm incision near to the fundus of the gallbladder and below the right costal margin was made as previously described (5). An atraumatic grasper was used to pull the gallbladder out of the abdominal wall through the incision (Fig. 1A). At the same time, the sub-umbilical trocar was removed and the pneumoperitoneum was released. For TL-GPS, a four-port method was used similar to LC and the soft choledochoscope was used instead of the inflexible cholecystoscope (Fig. 1B). A 1.0-cm incision was made on the parietal wall of the gallbladder fundus, which could be adjusted according to the size of the calculus. The bile was suctioned and endoscopes were inserted into the gallbladder for further exploration with a perfusion pressure of 60 cm H_2_O. Calculi that were ≥0.5 cm in diameter were removed using the basket (Fig. 2A). If the calculi were <0.5 cm, the suction trunk was used to drain them out. The biopsy forceps were used for the removal of any polypi <0.3 cm in diameter, whereas pre-electric coagulation on the polypus pedicle was necessary when the polypus appeared to be ≥0.3 cm (Fig. 2B–C). Polypus samples were taken in slices for rapid intraoperative pathological diagnosis to exclude the existence of gallbladder carcinoma. Following calculus removal, further exploration was necessary until spurting bile from the opening of the cystic duct (Fig. 2D) could be observed.

## Results

The procedures were performed successfully by one medical team, including nine cases of conversion into LC. The mean operating time ranged from 38 to 125 min (mean time, 72 min). No bile duct injuries occurred intra-operatively and no bile leakage or intra-abdominal infection was observed postoperatively. Two patients were shown to have residual sedimental stone through ultrasonic examination. Three cases of incisional infection were noted in the patients undergoing LA-GPS. Typical types of calculi and the use of surgical equipment are shown in Fig. 2.

The follow-up time ranged from 3 to 54 months, during which time six recurrences were observed in two years postoperatively and four patients received LC. In the nine conversion cases, one subcostal incisional hernia was seen exhibiting a localized bulge with a diameter of 4.0 cm and two cases with a polypus in a borderline or malignant manner were followed up postoperatively without tumor recurrence at 24 and 36 months.

## Discussion

Based on an in-depth knowledge of gallbladder function (6,7), it is suggested that GPS could provide a favorable treatment option for cholelithiasis. As laparoendoscopic surgery has developed, L-GPS combined with biliary endoscopy has been proposed by several experts in China (8). Despite remaining controversial, L-GPS warrants a trial for the following reasons: i) Without L-GPS, LC is likely to be required due to the pathology and injury to gallbladder function; thus, earlier medical intervention is likely to avoid an acute attack and cholecystectomy; ii) earlier GPS could remove all calculi, preserve the function of the gallbladder and decrease the higher risk of cholangiolithiasis and colon cancer following LC; and iii) in a number of European countries and China, there is a high proportion of cholesterol calculus cases due to a high-fat diet with limited exercise; these cases could be cured through an endoscopic approach, and the recurrence rate of such cholelithiasis could be reduced through modification of diet and living habits.

For patients who are to be offered GPS, indication judgments are of vital importance prior to surgery as there is still no criterion of absolute indications. Satisfactory gallbladder function of patients with cholelithiasis is essential since the preservation of a nonfunctional gallbladder is unhelpful and should be considered as a contraindication. An ultrasonic examination and ‘fat meal test’ (9) can assist with function evaluation. Patients with symptomatic cholelithiasis (gallstones combined with polypus) are strongly recommended for GPS, as are patients with a particular desire for the procedure; however, acute cholecystitis, histological carcinoma or cryptogenetic obstructive jaundice are incompatible and should be excluded from GPS indications (9,10). In the present study, 156 patients had suffered from acute episodes of biliary colic at least once and 68 patients had concurrent polypus. A total of 201 patients with non-specific upper abdominal discomfort and 153 patients who were asymptomatic were also suggested for GPS to prevent an acute episode or chronic injury to gallbladder function.

With regard to surgical approaches, LA-GPS and TL-GPS are both technically feasible. Even with extended exploration, no bile duct injury occurred during the surgeries in the present study. LA-GPS allows the gallbladder to be pulled out of the abdominal wall through a 2.0-cm subcostal incision (5); however, in the present study the procedure failed in eight patients, due to a probable carcinoma, and manipulation was confined to removing the calculus completely. In addition, TL-GPS was suspended for one patient due to an atrophic and nonfunctional gallbladder full of white bile. For these nine patients LC was then performed. A high preservation rate of 98.3% was obtained as a result of the strict indication checks; however, three cases of subcostal incisional infection occurred subsequent to LA-GPS, which were believed to be caused by spilt inflammatory bile. Following controlled antibiotics use and periodic dressing changes the incision healed well. One of the conversion cases, a male of morbid obesity, was noted to have an incisional hernia three months after the surgery: A longer subcostal incision was made because of the difficulties in gallbladder stretching and calculus removal prior to conversion into LC; in addition, intra-abdominal hypertension during LC may have led to muscle-slotting injuries that caused abdominal weakness.

These cases demonstrated the advantages of TL-GPS as it is not necessary to make a larger incision than the calculus size and the shorter contact period of spilt bile with the incision reduces the risk of incisional infection. Furthermore, in TL-GPS a titanium clamp can be used to temporarily obstruct the bile flow at the cystic duct to prevent calculus migration to the extrahepatic bile ducts and incarceration at the neck of the gallbladder or the bile duct opening. When complicated with extrahepatic bile duct calculus, the simultaneous laparoscopic common bile duct exploration can be performed, combined with fibercholedochoscope.

Calculus residue is considered the primary cause of cholelithiasis recurrence (11); thus, a follow-up was carried out for the observation of therapeutic effectiveness. In 517 patients, all macroscopic calculi and mucosal lesions were removed. Only two patients were shown to have sedimental residue on the third postoperative day under ultrasonic examination, without any recurrence in three months postoperatively. During long-term follow-up, six patients had a recurrence, with two cases of a single stone and four cases of several stones (recurrence rate, 1.2%). Three of the patients showed symptoms of cholecystitis; LC was performed on four patients. Application of the endoscope can avoid the limitations of one surgeon’s subjective perception, which may lead to the ignoring of tiny calculi. A magnified visual field and guidance on the screen can provide precise information on calculus size, number, location, appearance and characteristics, which are of great significance. Pressure homeostasis of cholecystoscopy and the use of a temporary obstruction of the cystic duct under the laparoscope may also avoid calculus migration and reduce the incidence of residues remaining. Combined with regular oral medication, such as ursodeoxycholic acid, following surgery, L-GPS can potentially cure cholelithiasis, with minor residue and recurrence rates.

In conclusion, the adoption of GPS reflects the important application of minimally invasive endoscopic surgery in treating biliary diseases. GPS provides a complementary approach to cure cholelithiasis. L-GPS is effective and TL-GPS is feasible for cholelithiasis and is particularly favorable in the prevention of incisional complications.
